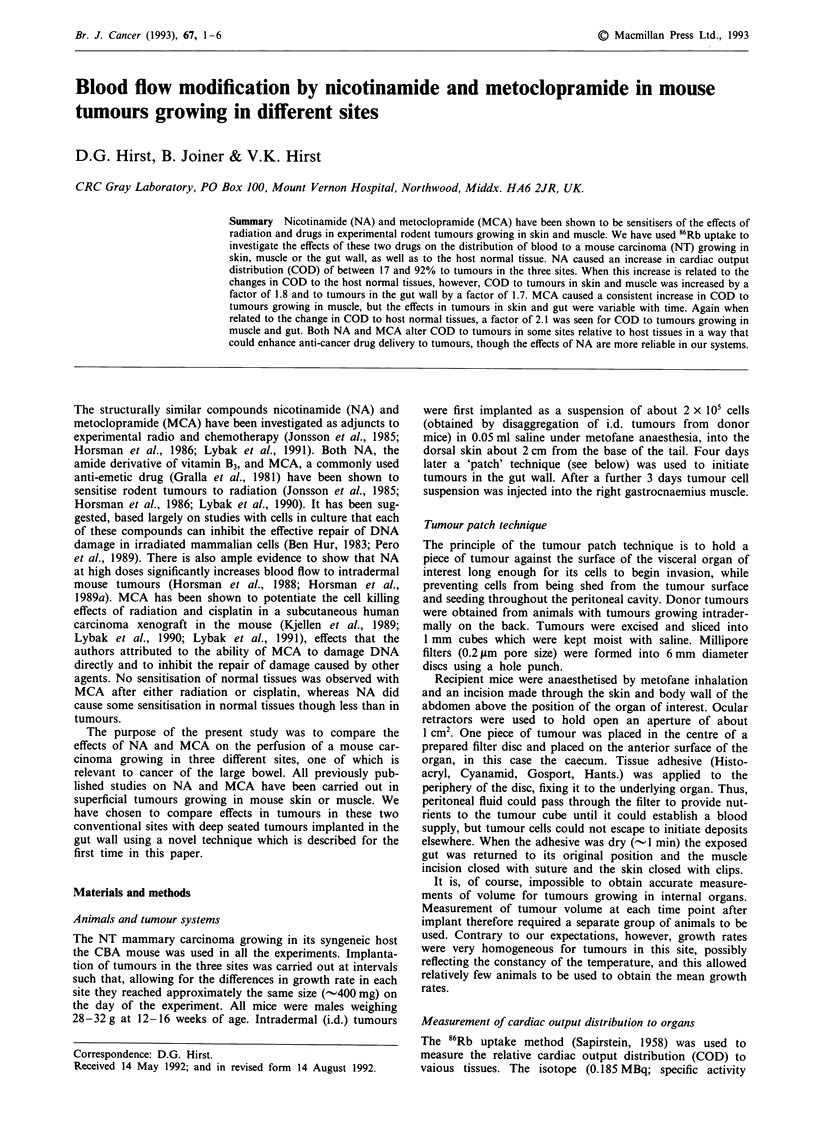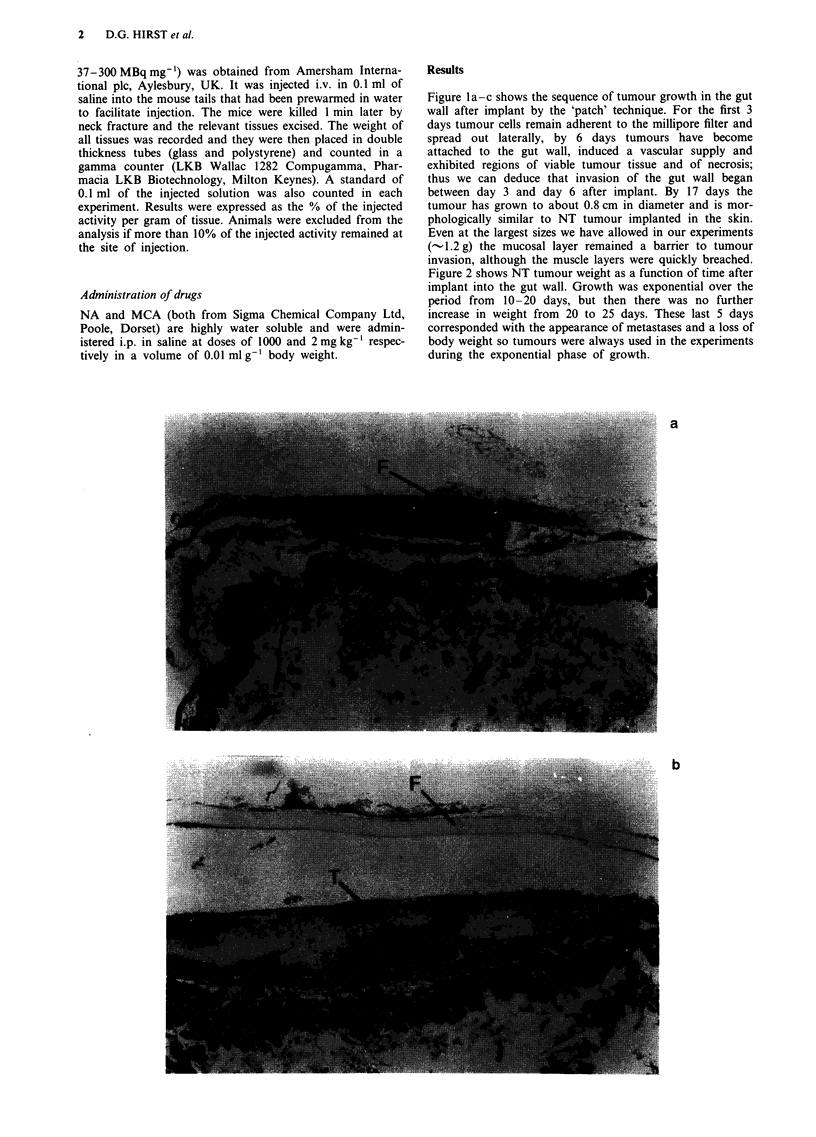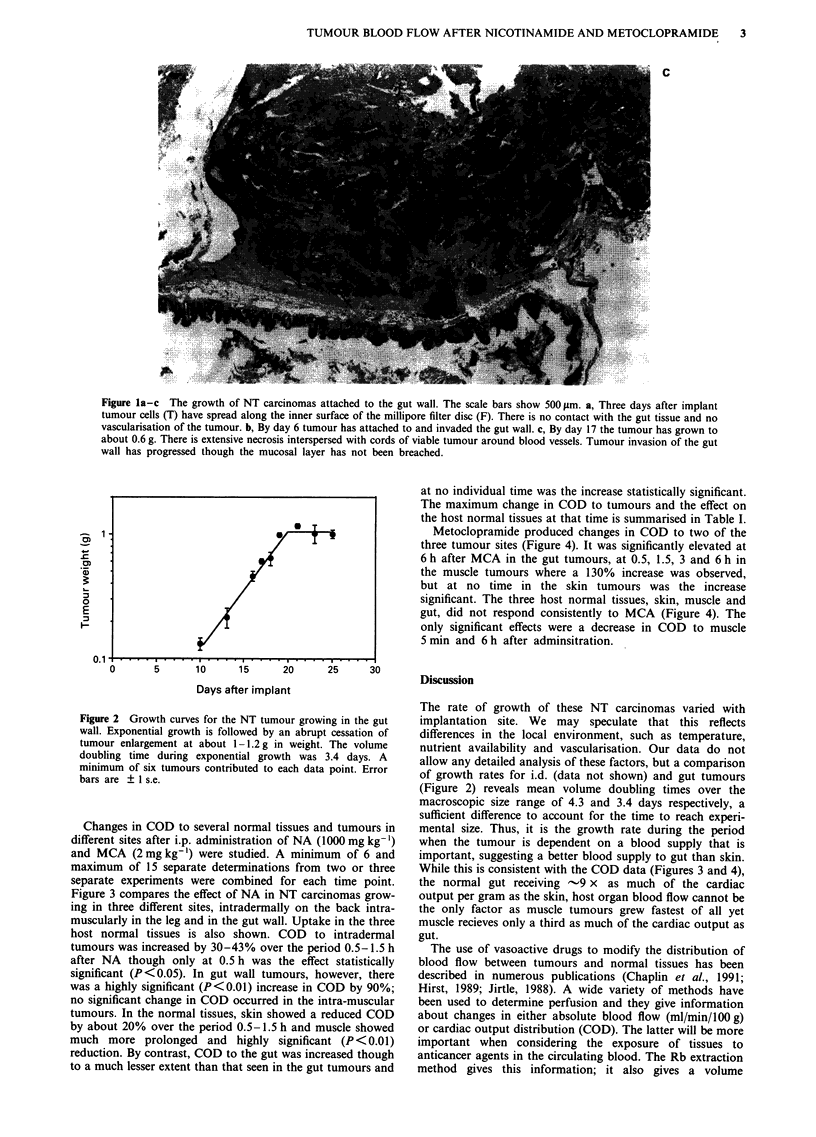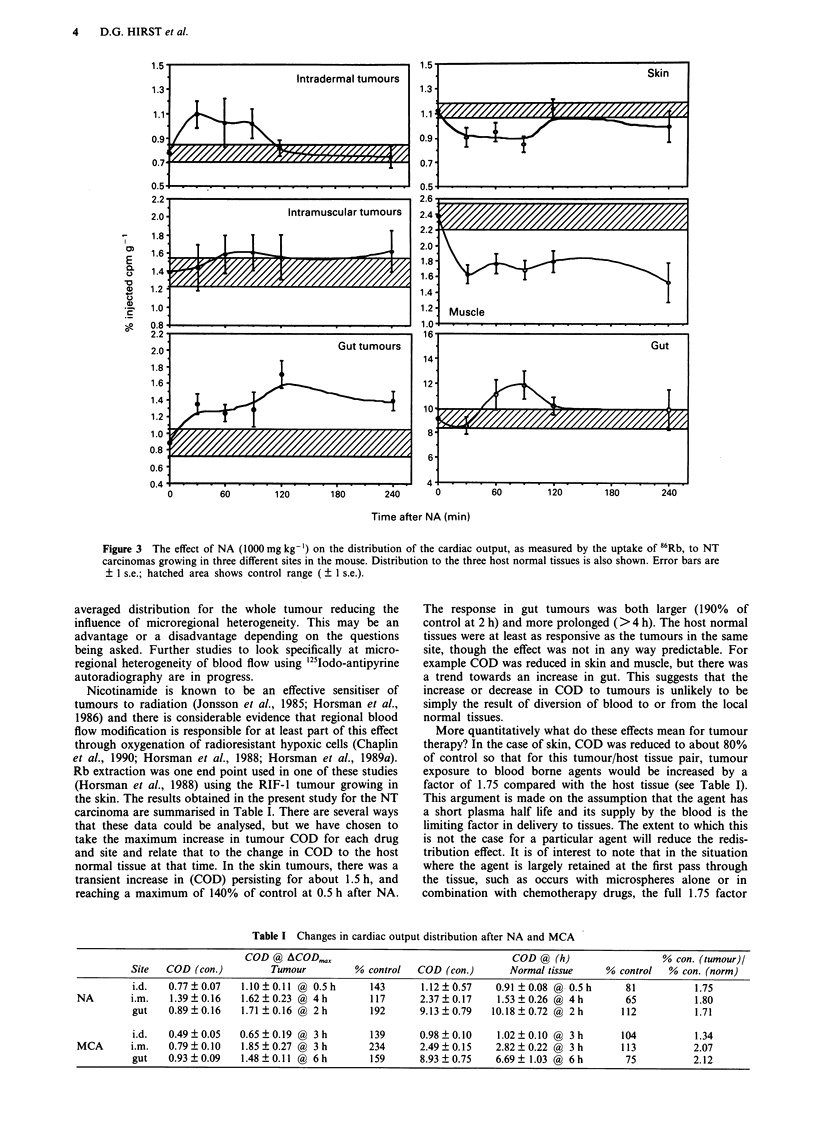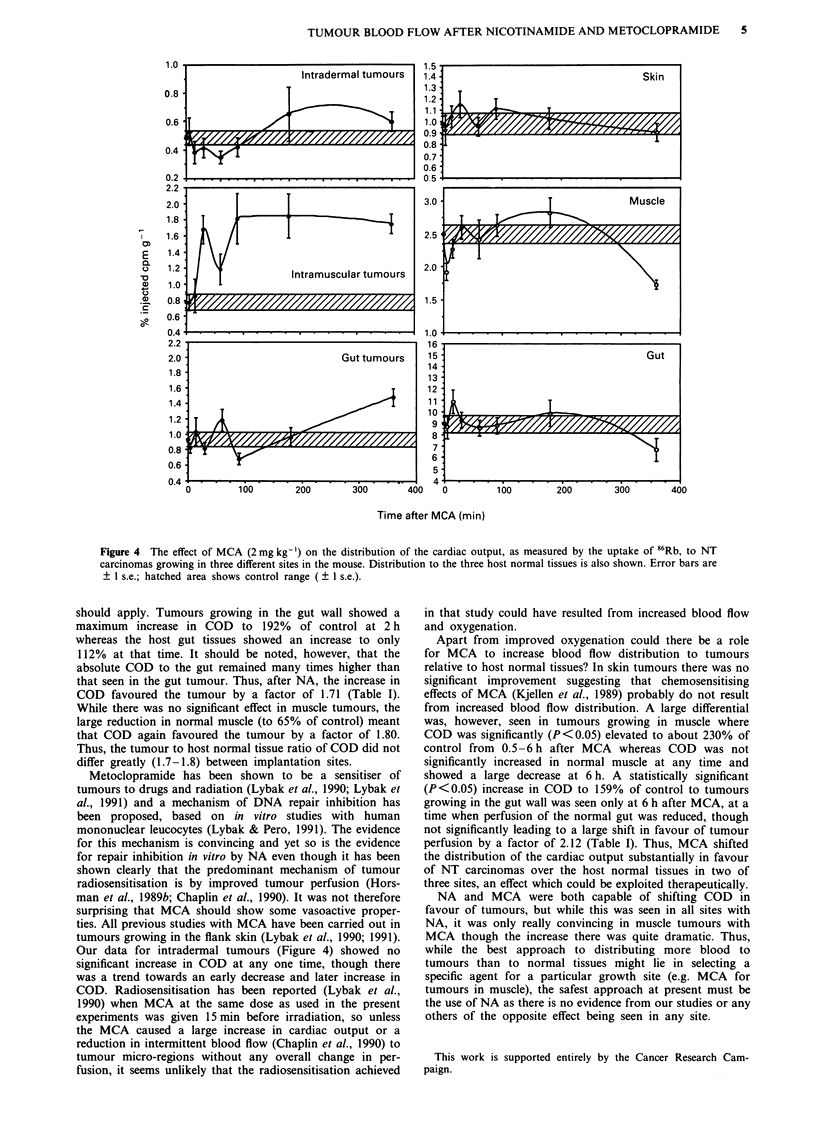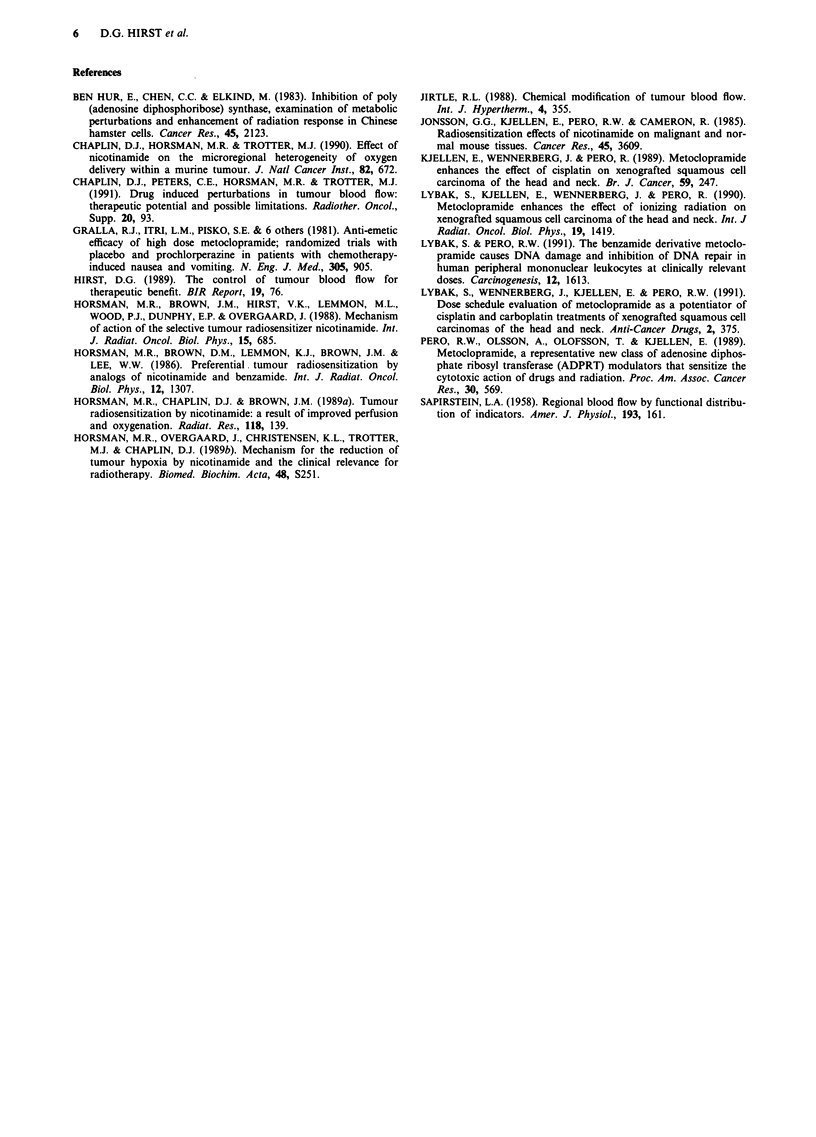# Blood flow modification by nicotinamide and metoclopramide in mouse tumours growing in different sites.

**DOI:** 10.1038/bjc.1993.1

**Published:** 1993-01

**Authors:** D. G. Hirst, B. Joiner, V. K. Hirst

**Affiliations:** CRC Gray Laboratory, Mount Vernon Hospital, Northwood, Middx. UK.

## Abstract

**Images:**


Br. J. Cancer (1993), 67, 1–6 © Macmillan Press Ltd., 1993

### Blood flow modification by nicotinamide and metoclopramide in mouse tumours growing in different sites

D.G. Hirst, B. Joiner & V.K. Hirst

*CRC Gray Laboratory, PO Box 100, Mount Vernon Hospital, Northwood, Middx. HA6 2JR, UK.*

**Summary** Nicotinamide (NA) and metoclopramide (MCA) have been shown to be sensitisers of the effects of radiation and drugs in experimental rodent tumours growing in skin and muscle. We have used $^{86}$Rb uptake to investigate the effects of these two drugs on the distribution of blood to a mouse carcinoma (NT) growing in skin, muscle or the gut wall, as well as to the host normal tissue. NA caused an increase in cardiac output distribution (COD) of between 17 and 92% to tumours in the three sites. When this increase is related to the changes in COD to the host normal tissues, however, COD to tumours in skin and muscle was increased by a factor of 1.8 and to tumours in the gut wall by a factor of 1.7. MCA caused a consistent increase in COD to tumours growing in muscle, but the effects in tumours in skin and gut were variable with time. Again when related to the change in COD to host normal tissues, a factor of 2.1 was seen for COD to tumours growing in muscle and gut. Both NA and MCA alter COD to tumours in some sites relative to host tissues in a way that could enhance anti-cancer drug delivery to tumours, though the effects of NA are more reliable in our systems.

The structurally similar compounds nicotinamide (NA) and metoclopramide (MCA) have been investigated as adjuncts to experimental radio and chemotherapy (Jonsson *et al.*, 1985; Horsman *et al.*, 1986; Lybak *et al.*, 1991). Both NA, the amide derivative of vitamin $B_3$, and MCA, a commonly used anti-emetic drug (Gralla *et al.*, 1981) have been shown to sensitise rodent tumours to radiation (Jonsson *et al.*, 1985; Horsman *et al.*, 1986; Lybak *et al.*, 1990). It has been suggested, based largely on studies with cells in culture that each of these compounds can inhibit the effective repair of DNA damage in irradiated mammalian cells (Ben Hur, 1983; Pero *et al.*, 1989). There is also ample evidence to show that NA at high doses significantly increases blood flow to intradermal mouse tumours (Horsman *et al.*, 1988; Horsman *et al.*, 1989*a*). MCA has been shown to potentiate the cell killing effects of radiation and cisplatin in a subcutaneous human carcinoma xenograft in the mouse (Kjellen *et al.*, 1989; Lybak *et al.*, 1990; Lybak *et al.*, 1991), effects that the authors attributed to the ability of MCA to damage DNA directly and to inhibit the repair of damage caused by other agents. No sensitisation of normal tissues was observed with MCA after either radiation or cisplatin, whereas NA did cause some sensitisation in normal tissues though less than in tumours.

The purpose of the present study was to compare the effects of NA and MCA on the perfusion of a mouse carcinoma growing in three different sites, one of which is relevant to cancer of the large bowel. All previously published studies on NA and MCA have been carried out in superficial tumours growing in mouse skin or muscle. We have chosen to compare effects in tumours in these two conventional sites with deep seated tumours implanted in the gut wall using a novel technique which is described for the first time in this paper.

### Materials and methods

### *Animals and tumour systems*

The NT mammary carcinoma growing in its syngeneic host the CBA mouse was used in all the experiments. Implantation of tumours in the three sites was carried out at intervals such that, allowing for the differences in growth rate in each site they reached approximately the same size (~400 mg) on the day of the experiment. All mice were males weighing 28–32 g at 12–16 weeks of age. Intradermal (i.d.) tumours were first implanted as a suspension of about $2 \times 10^5$ cells (obtained by disaggregation of i.d. tumours from donor mice) in 0.05 ml saline under metofane anaesthesia, into the dorsal skin about 2 cm from the base of the tail. Four days later a 'patch' technique (see below) was used to initiate tumours in the gut wall. After a further 3 days tumour cell suspension was injected into the right gastrocnaemius muscle.

### *Tumour patch technique*

The principle of the tumour patch technique is to hold a piece of tumour against the surface of the visceral organ of interest long enough for its cells to begin invasion, while preventing cells from being shed from the tumour surface and seeding throughout the peritoneal cavity. Donor tumours were obtained from animals with tumours growing intradermally on the back. Tumours were excised and sliced into 1 mm cubes which were kept moist with saline. Millipore filters (0.2 μm pore size) were formed into 6 mm diameter discs using a hole punch.

Recipient mice were anaesthetised by metofane inhalation and an incision made through the skin and body wall of the abdomen above the position of the organ of interest. Ocular retractors were used to hold open an aperture of about 1 cm$^2$. One piece of tumour was placed in the centre of a prepared filter disc and placed on the anterior surface of the organ, in this case the caecum. Tissue adhesive (Histoacryl, Cyanamid, Gosport, Hants.) was applied to the periphery of the disc, fixing it to the underlying organ. Thus, peritoneal fluid could pass through the filter to provide nutrients to the tumour cube until it could establish a blood supply, but tumour cells could not escape to initiate deposits elsewhere. When the adhesive was dry (~1 min) the exposed gut was returned to its original position and the muscle incision closed with suture and the skin closed with clips.

It is, of course, impossible to obtain accurate measurements of volume for tumours growing in internal organs. Measurement of tumour volume at each time point after implant therefore required a separate group of animals to be used. Contrary to our expectations, however, growth rates were very homogeneous for tumours in this site, possibly reflecting the constancy of the temperature, and this allowed relatively few animals to be used to obtain the mean growth rates.

### *Measurement of cardiac output distribution to organs*

The $^{86}$Rb uptake method (Sapirstein, 1958) was used to measure the relative cardiac output distribution (COD) to vaious tissues. The isotope (0.185 MBq; specific activity

Correspondence: D.G. Hirst.
Received 14 May 1992; and in revised form 14 August 1992.

37–300 MBq mg$^{-1}$) was obtained from Amersham International plc, Aylesbury, UK. It was injected i.v. in 0.1 ml of saline into the mouse tails that had been prewarmed in water to facilitate injection. The mice were killed 1 min later by neck fracture and the relevant tissues excised. The weight of all tissues was recorded and they were then placed in double thickness tubes (glass and polystyrene) and counted in a gamma counter (LKB Wallac 1282 Compugamma, Pharmacia LKB Biotechnology, Milton Keynes). A standard of 0.1 ml of the injected solution was also counted in each experiment. Results were expressed as the % of the injected activity per gram of tissue. Animals were excluded from the analysis if more than 10% of the injected activity remained at the site of injection.

*Administration of drugs*

NA and MCA (both from Sigma Chemical Company Ltd, Poole, Dorset) are highly water soluble and were administered i.p. in saline at doses of 1000 and 2 mg kg$^{-1}$ respectively in a volume of 0.01 ml g$^{-1}$ body weight.

### Results

Figure 1a–c shows the sequence of tumour growth in the gut wall after implant by the 'patch' technique. For the first 3 days tumour cells remain adherent to the millipore filter and spread out laterally, by 6 days tumours have become attached to the gut wall, induced a vascular supply and exhibited regions of viable tumour tissue and of necrosis; thus we can deduce that invasion of the gut wall began between day 3 and day 6 after implant. By 17 days the tumour has grown to about 0.8 cm in diameter and is morphologically similar to NT tumour implanted in the skin. Even at the largest sizes we have allowed in our experiments (~1.2 g) the mucosal layer remained a barrier to tumour invasion, although the muscle layers were quickly breached. Figure 2 shows NT tumour weight as a function of time after implant into the gut wall. Growth was exponential over the period from 10–20 days, but then there was no further increase in weight from 20 to 25 days. These last 5 days corresponded with the appearance of metastases and a loss of body weight so tumours were always used in the experiments during the exponential phase of growth.

a b

**Figure 1a–c** The growth of NT carcinomas attached to the gut wall. The scale bars show 500 μm. **a**, Three days after implant tumour cells (T) have spread along the inner surface of the millipore filter disc (F). There is no contact with the gut tissue and no vascularisation of the tumour. **b**, By day 6 tumour has attached to and invaded the gut wall. **c**, By day 17 the tumour has grown to about 0.6 g. There is extensive necrosis interspersed with cords of viable tumour around blood vessels. Tumour invasion of the gut wall has progressed though the mucosal layer has not been breached.

**Figure 2** Growth curves for the NT tumour growing in the gut wall. Exponential growth is followed by an abrupt cessation of tumour enlargement at about 1–1.2 g in weight. The volume doubling time during exponential growth was 3.4 days. A minimum of six tumours contributed to each data point. Error bars are ± 1 s.e.

Changes in COD to several normal tissues and tumours in different sites after i.p. administration of NA (1000 mg $kg^{-1}$) and MCA (2 mg $kg^{-1}$) were studied. A minimum of 6 and maximum of 15 separate determinations from two or three separate experiments were combined for each time point. Figure 3 compares the effect of NA in NT carcinomas growing in three different sites, intradermally on the back intramuscularly in the leg and in the gut wall. Uptake in the three host normal tissues is also shown. COD to intradermal tumours was increased by 30–43% over the period 0.5–1.5 h after NA though only at 0.5 h was the effect statistically significant ($P<0.05$). In gut wall tumours, however, there was a highly significant ($P<0.01$) increase in COD by 90%; no significant change in COD occurred in the intra-muscular tumours. In the normal tissues, skin showed a reduced COD by about 20% over the period 0.5–1.5 h and muscle showed much more prolonged and highly significant ($P<0.01$) reduction. By contrast, COD to the gut was increased though to a much lesser extent than that seen in the gut tumours and at no individual time was the increase statistically significant. The maximum change in COD to tumours and the effect on the host normal tissues at that time is summarised in Table I.

Metoclopramide produced changes in COD to two of the three tumour sites (Figure 4). It was significantly elevated at 6 h after MCA in the gut tumours, at 0.5, 1.5, 3 and 6 h in the muscle tumours where a 130% increase was observed, but at no time in the skin tumours was the increase significant. The three host normal tissues, skin, muscle and gut, did not respond consistently to MCA (Figure 4). The only significant effects were a decrease in COD to muscle 5 min and 6 h after adminsitration.

### Discussion

The rate of growth of these NT carcinomas varied with implantation site. We may speculate that this reflects differences in the local environment, such as temperature, nutrient availability and vascularisation. Our data do not allow any detailed analysis of these factors, but a comparison of growth rates for i.d. (data not shown) and gut tumours (Figure 2) reveals mean volume doubling times over the macroscopic size range of 4.3 and 3.4 days respectively, a sufficient difference to account for the time to reach experimental size. Thus, it is the growth rate during the period when the tumour is dependent on a blood supply that is important, suggesting a better blood supply to gut than skin. While this is consistent with the COD data (Figures 3 and 4), the normal gut receiving ~9 × as much of the cardiac output per gram as the skin, host organ blood flow cannot be the only factor as muscle tumours grew fastest of all yet muscle recieves only a third as much of the cardiac output as gut.

The use of vasoactive drugs to modify the distribution of blood flow between tumours and normal tissues has been described in numerous publications (Chaplin *et al.*, 1991; Hirst, 1989; Jirtle, 1988). A wide variety of methods have been used to determine perfusion and they give information about changes in either absolute blood flow (ml/min/100 g) or cardiac output distribution (COD). The latter will be more important when considering the exposure of tissues to anticancer agents in the circulating blood. The Rb extraction method gives this information; it also gives a volume

**Figure 3** The effect of NA (1000 mg kg$^{-1}$) on the distribution of the cardiac output, as measured by the uptake of $^{86}$Rb, to NT carcinomas growing in three different sites in the mouse. Distribution to the three host normal tissues is also shown. Error bars are ± 1 s.e.; hatched area shows control range ( ± 1 s.e.).

averaged distribution for the whole tumour reducing the influence of microregional heterogeneity. This may be an advantage or a disadvantage depending on the questions being asked. Further studies to look specifically at microregional heterogeneity of blood flow using $^{125}$Iodo-antipyrine autoradiography are in progress.

Nicotinamide is known to be an effective sensitiser of tumours to radiation (Jonsson *et al.*, 1985; Horsman *et al.*, 1986) and there is considerable evidence that regional blood flow modification is responsible for at least part of this effect through oxygenation of radioresistant hypoxic cells (Chaplin *et al.*, 1990; Horsman *et al.*, 1988; Horsman *et al.*, 1989*a*). Rb extraction was one end point used in one of these studies (Horsman *et al.*, 1988) using the RIF-1 tumour growing in the skin. The results obtained in the present study for the NT carcinoma are summarised in Table I. There are several ways that these data could be analysed, but we have chosen to take the maximum increase in tumour COD for each drug and site and relate that to the change in COD to the host normal tissue at that time. In the skin tumours, there was a transient increase in (COD) persisting for about 1.5 h, and reaching a maximum of 140% of control at 0.5 h after NA. The response in gut tumours was both larger (190% of control at 2 h) and more prolonged (>4 h). The host normal tissues were at least as responsive as the tumours in the same site, though the effect was not in any way predictable. For example COD was reduced in skin and muscle, but there was a trend towards an increase in gut. This suggests that the increase or decrease in COD to tumours is unlikely to be simply the result of diversion of blood to or from the local normal tissues.

More quantitatively what do these effects mean for tumour therapy? In the case of skin, COD was reduced to about 80% of control so that for this tumour/host tissue pair, tumour exposure to blood borne agents would be increased by a factor of 1.75 compared with the host tissue (see Table I). This argument is made on the assumption that the agent has a short plasma half life and its supply by the blood is the limiting factor in delivery to tissues. The extent to which this is not the case for a particular agent will reduce the redistribution effect. It is of interest to note that in the situation where the agent is largely retained at the first pass through the tissue, such as occurs with microspheres alone or in combination with chemotherapy drugs, the full 1.75 factor

**Table I** Changes in cardiac output distribution after NA and MCA

| | *Site* | *COD (con.)* | *COD @ $\Delta COD_{max}$ Tumour* | *% control* | *COD (con.)* | *COD @ (h) Normal tissue* | *% control* | *% con. (tumour)/ % con. (norm)* |
|---|---|---|---|---|---|---|---|---|
| | i.d. | 0.77 ± 0.07 | 1.10 ± 0.11 @ 0.5 h | 143 | 1.12 ± 0.57 | 0.91 ± 0.08 @ 0.5 h | 81 | 1.75 |
| NA | i.m. | 1.39 ± 0.16 | 1.62 ± 0.23 @ 4 h | 117 | 2.37 ± 0.17 | 1.53 ± 0.26 @ 4 h | 65 | 1.80 |
| | gut | 0.89 ± 0.16 | 1.71 ± 0.16 @ 2 h | 192 | 9.13 ± 0.79 | 10.18 ± 0.72 @ 2 h | 112 | 1.71 |
| | i.d. | 0.49 ± 0.05 | 0.65 ± 0.19 @ 3 h | 139 | 0.98 ± 0.10 | 1.02 ± 0.10 @ 3 h | 104 | 1.34 |
| MCA | i.m. | 0.79 ± 0.10 | 1.85 ± 0.27 @ 3 h | 234 | 2.49 ± 0.15 | 2.82 ± 0.22 @ 3 h | 113 | 2.07 |
| | gut | 0.93 ± 0.09 | 1.48 ± 0.11 @ 6 h | 159 | 8.93 ± 0.75 | 6.69 ± 1.03 @ 6 h | 75 | 2.12 |

**Figure 4** The effect of MCA (2 mg kg$^{-1}$) on the distribution of the cardiac output, as measured by the uptake of $^{86}$Rb, to NT carcinomas growing in three different sites in the mouse. Distribution to the three host normal tissues is also shown. Error bars are ± 1 s.e.; hatched area shows control range ( ± 1 s.e.).

should apply. Tumours growing in the gut wall showed a maximum increase in COD to 192% of control at 2 h whereas the host gut tissues showed an increase to only 112% at that time. It should be noted, however, that the absolute COD to the gut remained many times higher than that seen in the gut tumour. Thus, after NA, the increase in COD favoured the tumour by a factor of 1.71 (Table I). While there was no significant effect in muscle tumours, the large reduction in normal muscle (to 65% of control) meant that COD again favoured the tumour by a factor of 1.80. Thus, the tumour to host normal tissue ratio of COD did not differ greatly (1.7–1.8) between implantation sites.

Metoclopramide has been shown to be a sensitiser of tumours to drugs and radiation (Lybak *et al.*, 1990; Lybak *et al.*, 1991) and a mechanism of DNA repair inhibition has been proposed, based on *in vitro* studies with human mononuclear leucocytes (Lybak & Pero, 1991). The evidence for this mechanism is convincing and yet so is the evidence for repair inhibition *in vitro* by NA even though it has been shown clearly that the predominant mechanism of tumour radiosensitisation is by improved tumour perfusion (Horsman *et al.*, 1989*b*; Chaplin *et al.*, 1990). It was not therefore surprising that MCA should show some vasoactive properties. All previous studies with MCA have been carried out in tumours growing in the flank skin (Lybak *et al.*, 1990; 1991). Our data for intradermal tumours (Figure 4) showed no significant increase in COD at any one time, though there was a trend towards an early decrease and later increase in COD. Radiosensitisation has been reported (Lybak *et al.*, 1990) when MCA at the same dose as used in the present experiments was given 15 min before irradiation, so unless the MCA caused a large increase in cardiac output or a reduction in intermittent blood flow (Chaplin *et al.*, 1990) to tumour micro-regions without any overall change in perfusion, it seems unlikely that the radiosensitisation achieved in that study could have resulted from increased blood flow and oxygenation.

Apart from improved oxygenation could there be a role for MCA to increase blood flow distribution to tumours relative to host normal tissues? In skin tumours there was no significant improvement suggesting that chemosensitising effects of MCA (Kjellen *et al.*, 1989) probably do not result from increased blood flow distribution. A large differential was, however, seen in tumours growing in muscle where COD was significantly ($P<0.05$) elevated to about 230% of control from 0.5–6 h after MCA whereas COD was not significantly increased in normal muscle at any time and showed a large decrease at 6 h. A statistically significant ($P<0.05$) increase in COD to 159% of control to tumours growing in the gut wall was seen only at 6 h after MCA, at a time when perfusion of the normal gut was reduced, though not significantly leading to a large shift in favour of tumour perfusion by a factor of 2.12 (Table I). Thus, MCA shifted the distribution of the cardiac output substantially in favour of NT carcinomas over the host normal tissues in two of three sites, an effect which could be exploited therapeutically.

NA and MCA were both capable of shifting COD in favour of tumours, but while this was seen in all sites with NA, it was only really convincing in muscle tumours with MCA though the increase there was quite dramatic. Thus, while the best approach to distributing more blood to tumours than to normal tissues might lie in selecting a specific agent for a particular growth site (e.g. MCA for tumours in muscle), the safest approach at present must be the use of NA as there is no evidence from our studies or any others of the opposite effect being seen in any site.

This work is supported entirely by the Cancer Research Campaign.

### References

BEN HUR, E., CHEN, C.C. & ELKIND, M. (1983). Inhibition of poly (adenosine diphosphoribose) synthase, examination of metabolic perturbations and enhancement of radiation response in Chinese hamster cells. *Cancer Res.*, **45**, 2123.

CHAPLIN, D.J., HORSMAN, M.R. & TROTTER, M.J. (1990). Effect of nicotinamide on the microregional heterogeneity of oxygen delivery within a murine tumour. *J. Natl Cancer Inst.*, **82**, 672.

CHAPLIN, D.J., PETERS, C.E., HORSMAN, M.R. & TROTTER, M.J. (1991). Drug induced perturbations in tumour blood flow: therapeutic potential and possible limitations. *Radiother. Oncol.*, Supp. **20**, 93.

GRALLA, R.J., ITRI, L.M., PISKO, S.E. & 6 others (1981). Anti-emetic efficacy of high dose metoclopramide; randomized trials with placebo and prochlorperazine in patients with chemotherapy-induced nausea and vomiting. *N. Eng. J. Med.*, **305**, 905.

HIRST, D.G. (1989). The control of tumour blood flow for therapeutic benefit. *BIR Report*, **19**, 76.

HORSMAN, M.R., BROWN, J.M., HIRST, V.K., LEMMON, M.L., WOOD, P.J., DUNPHY, E.P. & OVERGAARD, J. (1988). Mechanism of action of the selective tumour radiosensitizer nicotinamide. *Int. J. Radiat. Oncol. Biol. Phys.*, **15**, 685.

HORSMAN, M.R., BROWN, D.M., LEMMON, K.J., BROWN, J.M. & LEE, W.W. (1986). Preferential tumour radiosensitization by analogs of nicotinamide and benzamide. *Int. J. Radiat. Oncol. Biol. Phys.*, **12**, 1307.

HORSMAN, M.R., CHAPLIN, D.J. & BROWN, J.M. (1989*a*). Tumour radiosensitization by nicotinamide: a result of improved perfusion and oxygenation. *Radiat. Res.*, **118**, 139.

HORSMAN, M.R., OVERGAARD, J., CHRISTENSEN, K.L., TROTTER, M.J. & CHAPLIN, D.J. (1989*b*). Mechanism for the reduction of tumour hypoxia by nicotinamide and the clinical relevance for radiotherapy. *Biomed. Biochim. Acta*, **48**, S251.

JIRTLE, R.L. (1988). Chemical modification of tumour blood flow. *Int. J. Hypertherm.*, **4**, 355.

JONSSON, G.G., KJELLEN, E., PERO, R.W. & CAMERON, R. (1985). Radiosensitization effects of nicotinamide on malignant and normal mouse tissues. *Cancer Res.*, **45**, 3609.

KJELLEN, E., WENNERBERG, J. & PERO, R. (1989). Metoclopramide enhances the effect of cisplatin on xenografted squamous cell carcinoma of the head and neck. *Br. J. Cancer*, **59**, 247.

LYBAK, S., KJELLEN, E., WENNERBERG, J. & PERO, R. (1990). Metoclopramide enhances the effect of ionizing radiation on xenografted squamous cell carcinoma of the head and neck. *Int. J Radiat. Oncol. Biol. Phys.*, **19**, 1419.

LYBAK, S. & PERO, R.W. (1991). The benzamide derivative metoclopramide causes DNA damage and inhibition of DNA repair in human peripheral mononuclear leukocytes at clinically relevant doses. *Carcinogenesis*, **12**, 1613.

LYBAK, S., WENNERBERG, J., KJELLEN, E. & PERO, R.W. (1991). Dose schedule evaluation of metoclopramide as a potentiator of cisplatin and carboplatin treatments of xenografted squamous cell carcinomas of the head and neck. *Anti-Cancer Drugs*, **2**, 375.

PERO, R.W., OLSSON, A., OLOFSSON, T. & KJELLEN, E. (1989). Metoclopramide, a representative new class of adenosine diphosphate ribosyl transferase (ADPRT) modulators that sensitize the cytotoxic action of drugs and radiation. *Proc. Am. Assoc. Cancer Res.*, **30**, 569.

SAPIRSTEIN, L.A. (1958). Regional blood flow by functional distribution of indicators. *Amer. J. Physiol.*, **193**, 161.